# Let-7 microRNAs Are Possibly Associated with Perineural Invasion in Colorectal Cancer by Targeting IGF Axis

**DOI:** 10.3390/life12101638

**Published:** 2022-10-19

**Authors:** Andrei Marian Niculae, Maria Dobre, Vlad Herlea, Teodora Ecaterina Manuc, Bogdan Trandafir, Elena Milanesi, Mihail Eugen Hinescu

**Affiliations:** 1 Victor Babes National Institute of Pathology, 050096 Bucharest, Romania; 2 Faculty of Medicine, Carol Davila University of Medicine and Pharmacy, 050474 Bucharest, Romania; 3 Fundeni Clinical Institute, 022328 Bucharest, Romania

**Keywords:** insulin-like growth factor, let-7 microRNAs, colorectal cancer, perineural invasion

## Abstract

Increased insulin-like growth factor (IGF) axis activity is associated with the development and progression of different types of malignancies, including colorectal cancer (CRC). MicroRNAs (miRNAs) belonging to the let-7 family have been reported to target genes involved in this axis and are known as tumor suppressors. In this study, in silico bioinformatic analysis was performed to assess miRNA–mRNA interactions between eight miRNAs belonging to the let-7 family and genes involved in the IGF signaling pathway, coding for receptors and substrates. miRNAs’ expression analysis revealed that hsa-let-7a-5p, hsa-let-7b-5p, hsa-let-7c-5p, hsa-let- 97 7d-5p, hsa-let-7e-5p, hsa-let-7f-5p, and hsa-let-7g-5p were significantly down-regulated in 25 CRC tumoral tissues (T) compared to the corresponding adjacent peritumoral tissues (PT). Moreover, our results showed an upregulation of miR-let-7e-5p in CRC tissues with mutations in KRAS codon 12 or 13, and, for the first time, found a specific dysregulation of let-7a-5p, let-7b-5p, let-7c-5p, let-7d-5p, and let-7i-5p in CRC with perineural invasion. Our results sustain the relationship between the IGF axis, let-7 miRNAs, and CRC and suggest an association between the expression of these miRNAs and perineural invasion.

## 1. Introduction

The insulin and insulin-like growth factor (IGF) signaling system is one of the main actors of growth and energy metabolism and plays a significant role in the pathogenesis and progression of many cancers. Although insulin and IGF serve different physiological functions, they share signaling pathways that involve phosphoinositide 3-kinase (PI3K), Akt or Ras, and MAP kinase [1]. Through these pathways, they mediate responses to a variety of other cellular stimuli, promoting cell proliferation and inhibiting apoptosis [2]. Therefore, this axis is a potential target for multiple lines of therapy, being incriminated also in tumor resistance and invasiveness. Silencing the IGF pathway has shown promising results in preclinical trials, albeit with significant challenges [3,4].

IGFs are small peptides isolated in human plasma which were named due to their resemblance to proinsulin. IGF-1 is secreted by multiple organs and tissues and can act both as endocrine, exocrine, or autocrine hormones [5].

The connection between the IGF-1 signaling pathway and colorectal cancer (CRC) has been suspected more than 10 years due to observation of the association between CRC and lifestyle factors (mainly physical inactivity and obesity) mediated by insulin resistance and hyperinsulinemia via the IGF axis [6]. 

Notably, one of the hallmarks of CRC initiation and progression is the Warburg effect—enhanced glucose uptake and aerobic glycolysis even in the presence of mitochondria functioning. However, the exact mechanisms involved in this type of metabolic reprogramming are still unknown [7].

Studies in CRC patients have shown that plasma levels of IGF-1 are higher in CRC patients compared to healthy controls [8], but they are not correlated to disease burden or post-operative progression [4]. Moreover, in CRC tissues, a wide range of positivity of IGF-1, IGF-2, and IGF-1R [9,10,11,12] as well as an impairment of these transcript levels [9,11,13] have been found.

The let-7 mi-RNA family is known as a tumor suppressor, and some studies have proposed it as a biomarker and prognostic factor in multiple fields of oncology and other diseases [14]. IGF1/IGF1R are potentially targeted by the let-7 miRNA family. The liaison between let-7 family miRNAs, CRC, and IGF1 pathways has previously been reported [15]. However, the association between let-7 family miRNAs’ expression and mechanisms leading to perineural invasion has not been deeply studied.

Perineural invasion (PNI) is a possible course for metastatic spread in a variety of cancers, including CRC, and is usually a poor prognostic factor [16]. Moreover, PNI presence has been associated with a higher T and N stage, histological features of adenocarcinoma, and higher tumor grade [17].

In the last available review highlighting the connection between miRNAs and PNI in different malignancies, miR-128-3p, miR-3679-5p, and miR-145 were tied to PNI in CRC. Surprisingly, miRNAs belonging to the let-7 family have been associated with PNI in multiple other types of cancer [18], but no studies in CRC are reported. Evidently, research in this narrow field has been sparse.

This study started by bioinformatics analysis to assess the relationship between miRNAs hsa-let-7a-5p, hsa-let-7b-5p, hsa-let-7c-5p, hsa-let-7d-5p, hsa-let-7e-5p, hsa-let-7f-5p, hsa-let-7g-5p, hsa-let-7i-5p, and genes involved in the IGF1 signaling pathway, coding for receptors and substrates. This analysis was followed by the comparison of the expression level of these miRNAs in 25 CRC tumoral and in the corresponding adjacent peritumoral tissues. Furthermore, the association between miRNAs’ expression and tumoral features was evaluated. The results of this work confirmed the expression impairment of these miRNAs in CRC and suggested their association with perineural invasion.

## 2. Materials and Methods

### 2.1. Patients

Twenty-five patients with confirmed primary CRC who underwent curative surgical intervention at the Fundeni Clinical Institute, Bucharest, Romania were enrolled in the study. Tumoral (T) and the corresponding adjacent peritumoral mucosa (PT) tissues were collected from patients. The tissues were formalin fixed and paraffin embedded (FFPE) for histological, immunohistochemical evaluation and DNA isolation. Part of the T and PT tissue was preserved in RNA protect Tissue Reagent (Qiagen, Hilden, Germany) and then stored at −80 °C until RNA isolation. Informed consent was obtained from all of the patients, and the study was conducted in accordance with the Declaration of Helsinki and approved by the Ethics Committee of the “Fundeni” Clinical Institute (11 December 2019) and Victor Babes National Institute of Pathology (approval number 78 of 3 December 2019). In Table 1 the clinical features of the patients and the characteristics of the analyzed tumor specimens are presented.

### 2.2. KRAS/BRAF Mutation Detection and MSI Evaluation

DNA isolation from FFPE tissues and the detection of *KRAS* codon 12 and codon 13 mutations, as well as the evaluation of microsatellite instability (MSI), were performed as previously described [19]. *BRAF* mutations in codon 600 and codon 601 were detected using the BRAF 600/601 StripAssay (ViennaLab Diagnostic GmbH, Vienna, Austria) according to the manufacturer’s protocol.

### 2.3. In Silico let-7 miRNAs and IGF1 Signaling Pathway Interaction

The interactions between the miRNAs hsa-let-7a-5p, hsa-let-7b-5p, hsa-let-7c-5p, hsa-let-7d-5p, hsa-let-7e-5p, hsa-let-7f-5p, hsa-let-7g-5p, and hsa-let-7i-5p and the genes involved in the IGF1 signaling pathway, coding for receptors and substrates (*IGF1R*, *IGF2R*, *INSR*, *IRS1,* and *IRS2*) were identified using miRTargetLink 2.0 [20]. This tool provides miRNA–target interactions experimentally validated (weak and strong) and computationally predicted. 

### 2.4. miRNAs Expression Analysis

Total RNA, including miRNAs, was isolated using a miRNeasy Mini Kit (Qiagen, Hilden, Germany) according to the manufacturer’s protocol. The reverse transcription of 10 ng RNA was performed with a miRCURY LNA RT Kit (Qiagen), and the expression of the selected 8 miRNAs involved in the IGF-1 signaling pathway was evaluated using a miRCURY LNA SYBR Green PCR Kit and a miRCURY LNA miRNA PCR Assay (Qiagen). For each sample, two technical replicates were evaluated, and the difference in the Ct value between the duplicates was <0.4 cycles. The Ct data were normalized against the geometric mean of SNORD38B and SNORD49A. In the comparison of T vs. PT, the miRNA expression data are presented as 2 ^−∆∆Ct^ values (fold change—FC values) using the values of PT tissues as control. In the other comparisons, showing a stratification of the PT and T samples according to the presence of PNI and *KRAS* mutations, data are presented as 2 ^−∆Ct^ values.

### 2.5. Statistical Analysis

The non-parametric Wilcoxon signed-rank test was applied in order to assess the differences between paired tumoral and the adjacent peritumoral mucosa, since the values of miRNA levels were not normally distributed (Shapiro–Wilk test, *p* < 0.05). The Mann–Whitney test was used to compare the tumoral miRNAs levels in the other comparisons. The differences in miRNA levels between the groups were considered significant when *p* < 0.05 and 0.65 ≥ FC ≥ 1.5. The Statistical Package for the Social Sciences (SPSS version 20.0, IBM, New York, NY, USA) and GraphPad Prism 8.4.3 (GraphPad Software, San Diego, CA, USA). were used to perform statistical analysis and generate the graphs.

## 3. Results

**Figure 1:**
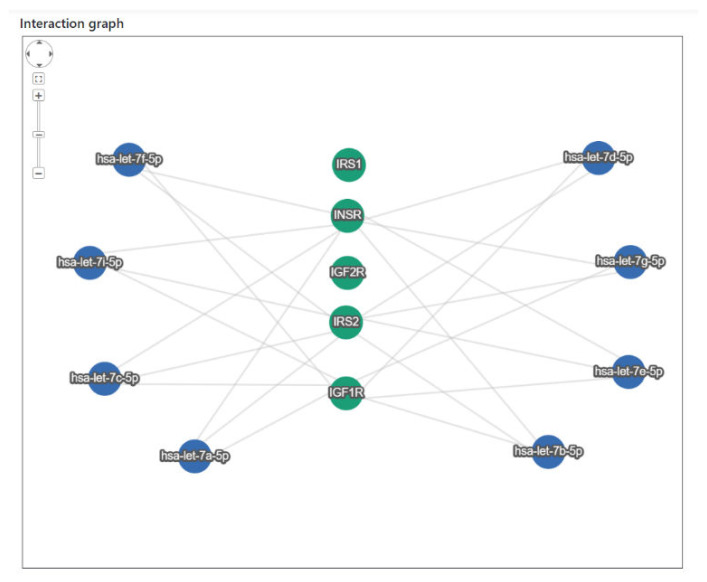
Interaction graph between let-7 miRNAs (blue nodes) and genes involved in the IGF1 signaling pathway (green nodes).

The in silico analysis to assess the miRNA–mRNA interaction between the eight let-7 miRNAs and the genes involved in the IGF1 signaling pathway, coding for receptors and substrates (IGF1R, IGF2R, INSR, IRS1, and IRS2), showed that all of the miRNAs were predicted or validated to target the selected genes, except IGF2R and IRS1. The interaction graph is reported in Figure 1, and the types of interactions (predicted or validated) are reported in supplementary data (Table S1).

**Figure 2:**
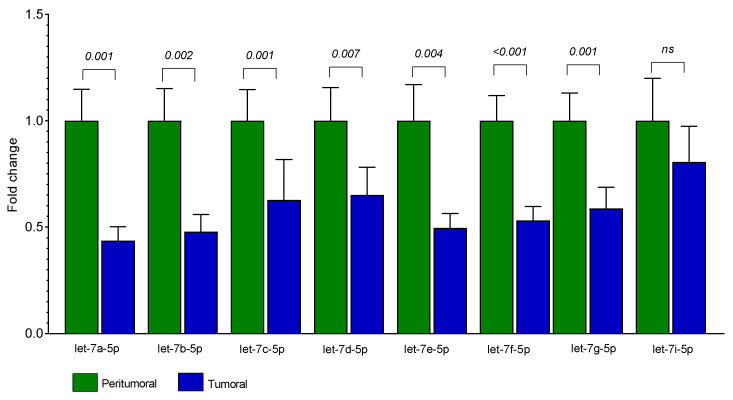
Expression levels of let-7 miRNAs in T and PT tissues from 25 patients with CRC. Bars represent the mean of expression ± SEM. Statistical significance was calculated with Wilcoxon signed-rank test; *ns* = not significant.

The differential expression between tumoral (T) and the paired adjacent peritumoral mucosa (PT) showed that seven out of the eight analyzed let-7 miRNAs were significantly downregulated in T: let-7a-5p (FC = 0.44, *p* = 0.001), let-7b-5p (FC = 0.48, *p* = 0.002), let-7c-5p (FC = 0.63, *p* = 0.001), let-7d-5p (FC = 0.65, *p* = 0.007), let-7e-5p (FC = 0.50, *p* = 0.004), let-7f-5p (FC = 0.53, *p* < 0.001), and let-7g-5p (FC = 0.59, *p* = 0.001). Let-7i-5p was moderately less expressed in T tissue without reaching statistical significance (FC = 0.80, *p* = 0.115) (Figure 2).

**Figure 3:**
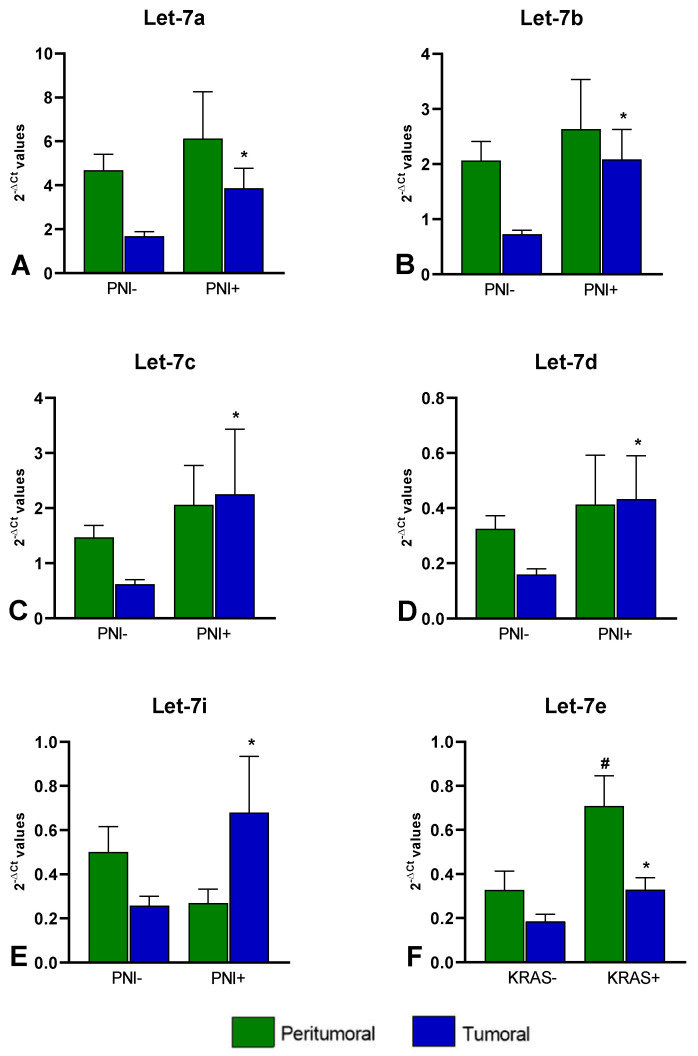
miRNAs differentially expressed between PNI+ and PNI- (**A**) let-7a-5p (FC = 2.30); (**B**) let-7b-5p (FC = 2.87); (**C**) let-7c-5p (FC = 3.65); (**D**) let-7d-5p (FC = 2.71); (**E**) and let-7i-5p (FC = 2.63); and miRNA differentially expressed between *KRAS*+ and *KRAS*- (**F**) let-7e (FC = 1.78 in T and FC = 2.15 in PT). Bars represent the mean of expression ± SEM. Statistical significance was calculated with Mann–Whitney test; * *p* < 0.05 in the comparisons performed in T; ^#^ *p* < 0.05 in the comparisons performed in PT.

Considering the comparison between tumoral tissues with perineural invasion (PNI+) vs. those without perineural invasion (PNI-), we found an upregulation in the PNI+ samples of the following miRNAs: let-7a-5p (FC = 2.30, *p* = 0.014), let-7b-5p (FC = 2.87, *p* = 0.006), let-7c-5p (FC = 3.65, *p* = 0.011), let-7d-5p (FC = 2.71, *p* = 0.009), and let-7i-5p (FC = 2.63, *p* = 0.036) (Figure 3A–E).

When comparing the miRNAs’ expression levels between the 12 T with KRAS mutation (codon 12 or codon 13) vs. the 13 T without KRAS mutation, we found that miR-let-7e-5p was upregulated (FC = 1.78, *p* = 0.040). This increase was also observed when comparing the PT tissues of the two groups (FC = 2.15; *p* = 0.004) even though none of the PT tissues presented mutations (Figure 3F).

A downregulation of miRNAs let-7c-5p, let-7d-5p, and let-7i-5p was found when comparing T with BRAF mutation vs. those without BRAF mutation (FC = 0.21 *p* = 0.027; FC = 0.32 *p* = 0.027; FC = 0.30 *p* = 0.040, respectively). This result cannot be suggestive, since only two T tissues reported BRAF mutation. 

No significant difference in miRNAs expression was observed in relation to the other characteristics of the tumors or patient features which includes localization, grade, histologic subtype, lymphovascular invasion, microsatellite instability, sex, family history of cancer, and reported moderate alcohol consumption (*p* > 0.05)

## 4. Discussion

Insulin and insulin-like growth factor 1 (IGF-1) act on the tyrosine kinase receptors INSR and IGF-1R. INSR and IGF-1R are frequently overexpressed in cancer cells [21,22], where they activate a variety of intracellular signaling cascades that inhibit apoptosis and promote cell cycle progression [22,23].

Studies conducted on colonic tissues aimed at the detection of the IGF-1R gene and protein expression, found that the IGF-1R mRNA levels are significantly higher in CRC tissue compared with adjacent normal mucosa [13,15]. Moreover, the level of IGF-1R protein is associated with tumor localization, grading, tumor growth, lymphatic vessel invasion, and mismatch repair protein expression status [12].

Investigations on INSR have shown that the isoform A is significantly higher in CRC than in normal tissues [24], while isoform B expression has been indicated to be reduced in adenomas compared to normal colon tissue [25].

Using bioinformatics analysis, we observed the miRNA–mRNA interaction between eight let-7 miRNAs and three genes coding for the receptors IGF-1R and INSR, and the insulin receptor substrate IRS2. Through qRT-PCR we found that let-7a-5p, let-7b-5p, let-7c-5p, let-7d-5p, let-7e-5p, let-7f-5p, and let-7g-5p were downregulated in the tumoral tissues. These results partially reproduced those obtained by Li and collaborators, which found a significant downregulation of let-7a, let-7b, and let-7e in CRC tissues, whereas no changes in the expression levels of let-7c, let-7d, let-7f, and let-7g were observed in their study [15]. Notably, in our study let-7a, let-7b, and let-7e were identified as the most downregulated miRNAs.

The involvement of the let-7 family in CRC has been investigated in other studies. Results on the expression of let-7a has indicated that this miRNA shows low expression in human CRC cell lines [26], and that its levels could be upregulated by cisatracurium [27], a compound which inhibits the proliferation and induces apoptosis of the cancer cells [28]. Studies in human tissues have indicated let-7a-5p to be downregulated in CRC compared to normal tissue from non-CRC controls [29,30], whereas no difference has been found in a study comparing let-7a-5p between adjacent normal and tumor tissues [31]. Interestingly, a negative correlation between this miRNA and tumor size, stage and lymph node metastasis in CRC patients has been observed [32].

In line with our results, a downregulation of let-7b was observed in tumoral tissues [15,33] and in the plasma [34] of CRC patients, and, along with a panel of other miRNAs, it has been indicated as a biomarker able to distinguish between normal and tumoral colonic tissues [35]. In contrast, in a study performed on FFPE samples, the expression of let-7b was found to be upregulated in CRC [29]. This difference can be explained by the fact that comparing miRNA expression profiles of paired fresh frozen and FFPE samples; only 27–38% of the differentially expressed miRNAs overlapped between the two source systems [36].

Let-7c has been indicated as a hub miRNA related to CRC prognosis [37], and, contrasting with our results, its level has been found to be increased in CRC tissue samples as compared to normal colonic tissues [29]. Literature data on the expression of let-7d-5p are also contrasting, with some works reporting its diminished level in CRC tissues [38] as in our study, and other studies detecting its upregulation in tumoral tissues [29] and cell lines [39]. Regarding the expression of let-7e-5p, a study on TCGA datasets found its expression levels to be lower in colorectal tumor tissues than in normal tissues [40], as confirmed by Li et al. and our data, which detected the same changes by qRT-PCR [15]. Finally, our study revealed the under-expression of two other miRNAs, let-7f-5p and let-7g-5p. The results on the involvement of these miRNAs in CRC are not clear, with one study showing let-7g-5p upregulation in tumoral tissues [29] and one, in agreement with our findings, showing decreased expression of let-7f in CRC cell lines [41].

The discrepancies among the studies on the expression of let-7 miRNAs in CRC could be due to the different characteristics of tumoral tissues in terms of the grade, stage, tumor locations, presence of perineural invasion, and *KRAS* or *BRAF* mutation status. Indeed, our results showed an upregulation of let-7e-5p in CRC tissues with mutation in *KRAS* codon 12 or 13 and, for the first time, suggested an association between let-7a-5p, let-7b-5p, let-7c-5p, let-7d-5p, and let-7i-5p and perineural invasion in CRC. Among the identified miRNAs, only let-7a and let-7d have so far been discussed in the literature as being involved in PNI, reporting a downregulation in oral squamous cell carcinoma [42] and an upregulation in head and neck squamous carcinoma [43] in PNI condition, respectively. Our study suggests a possible association between the let-7 miRNAs/IGF axis and PNI in CRC patients, without reporting functional validation. Due to the importance of PNI as a negative prognostic factor in CRC, further exploration in a larger cohort of patients is necessary, as well as functional studies to clarify the intricate mechanisms by which miRNAs are involved in PNI.

## 5. Conclusions

In conclusion, our results showed a general downregulation of let-7 miRNAs in tumoral CRC tissues. The let-7 miRNA profile in CRC is related to specific clinicopathological characteristics of tumors, mainly *KRAS* or *BRAF* mutations and the presence of PNI. These characteristics must be considered when conducting studies for the identification of miRNAs as prognostic and predictive biomarkers, as well as for the application of let-7-targeting therapy against CRC.

## Figures and Tables

**Table 1:** Clinical features of the patients and characteristics of the analyzed tumor specimens.

Features	CRC Patients (N = 25)
**Age (mean ± SD; min-max)**	65.56 ± 11.53 (30–83)
**Sex (N; %)**	F (13; 52%); M (12; 48%)
**Family history of cancer (N; %)**	6; 24%
**Moderate alcohol consumption (N; %)**	12; 48%
**Localization (N; %)**	Ascending colon (5; 20%) Transverse colon (2; 8%) Descending colon (3; 12%) Sigmoid (5; 20%) RSJ (6; 24%) Rectum (4; 16%)
**Grade (N; %)**	G1 (4; 16 %) G2 (14; 56%) G3 (7; 28%)
**TNM (N)**	T2 N0 M0 (N = 1); T2 N1 M0 (N = 1); T2 N2 M0 (N = 1); T3 N0 M0 (N = 10); T3 N1 M0 (N = 5); T3 N2 M0 (N = 3); T4 N0 M0 (N = 2); T4 N1 M0 (N = 1); T4 N2 M0 (N = 1);
**Histologic subtype (N; %)**	Adenocarcinoma (21; 84%) Mucinous (4; 16%)
**Lymphovascular invasion (N; %)**	9; 36%
**Perineural invasion (N; %)**	6; 24%
**Microsatellite instability (N; %)**	2; 8%
**KRAS/BRAF mutations**	
***KRAS* codon 12 (N; %)**	9; 36%
***KRAS* codon 13 (N; %)**	3; 12%
***BRAF* codon 600 (N; %)**	2; 8%

## Data Availability

The data presented in this study are available on reasonable request from the corresponding author.

## Supplementary Materials

The following are available online at https://www.mdpi.com/article/10.3390/life12101638/s1, Table S1: miRTargetLink 2.0 analysis showing the interaction between the analyzed miRNAs and IGF1R, IGF2R, INSR, IRS1, and IRS2 genes.
